# Supplementary material for: Increased Dynamics of α-Synuclein Fibrils by β-Synuclein Leads to Reduced Seeding and Cytotoxicity
Source: Sci Rep. 2019 Nov 26;9:17579. doi: 10.1038/s41598-019-54063-8 (PMC6879756; doi:10.1038/s41598-019-54063-8)
Supplement: Supplementary file 1 — Supplementary Information [file 41598_2019_54063_MOESM1_ESM.docx]

**Supplemental Information**

**Increased Dynamics of α**-**Synuclein Fibrils by β-Synuclein Leads to Reduced Seeding and Cytotoxicity**

Xue Yang^1$^, Jonathan K. Williams^1$^, Run Yan^2^, M. Maral Mouradian^2^, and Jean Baum^1*^

^1^Department of Chemistry and Chemical Biology, Rutgers University, Piscataway, New Jersey 08854

^2^RWJMS Institute for Neurological Therapeutics, Rutgers Biomedical and Health Sciences, and Department of Neurology, Robert Wood Johnson Medical School, Rutgers University, Piscataway, New Jersey, 08854

^$^These authors contributed equally to this work.


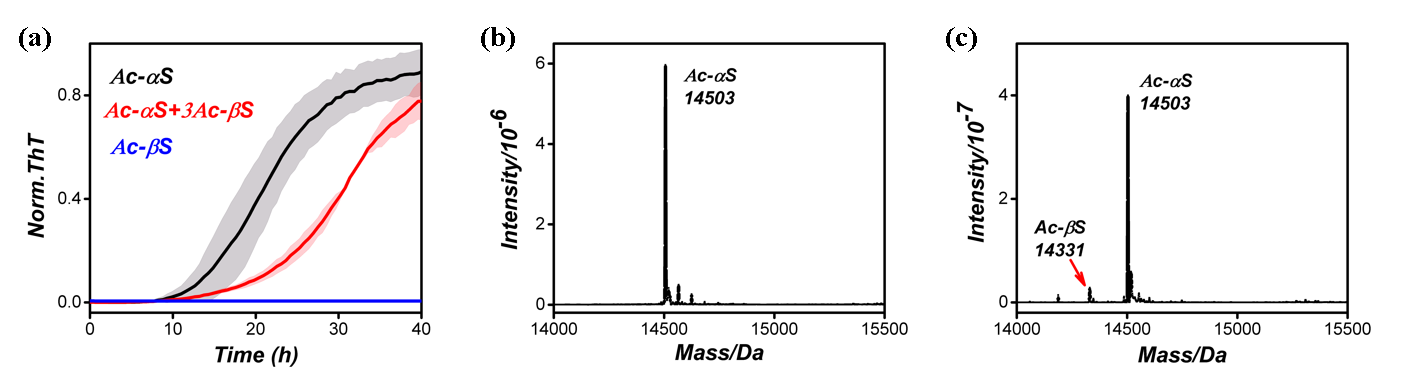


**Figure S1.** (a) Normalized change in ThT fluorescence signal of 70 µM Ac-αS (black), 210 µM Ac-βS (blue), or a mixture of 70 µM Ac-αS + 210 µM Ac-βS (1:3) (red) incubated at 37°C in 10 mM PBS with Teflon beads and shaking. (b,c) ESI-MS data showing the protein composition of αS fibrils and co-incubated αS/βS fibrils.


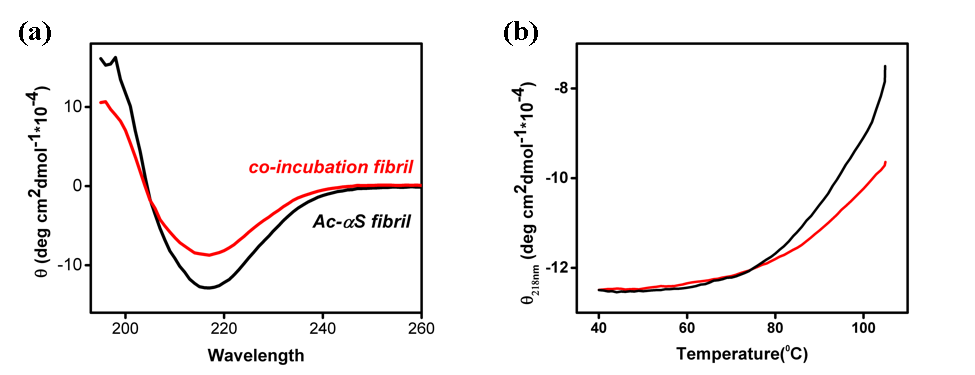


**Figure S2.** (a) Far-UV CD wavelength scan spectrum of αS fibril (black) and co-incubated αS/βS fibril (red) showing similar secondary structure. (b) Thermal stability curve of αS fibril (black) and co-incubation αS/βS fibril (red), monitored by CD signal change at 218nm.


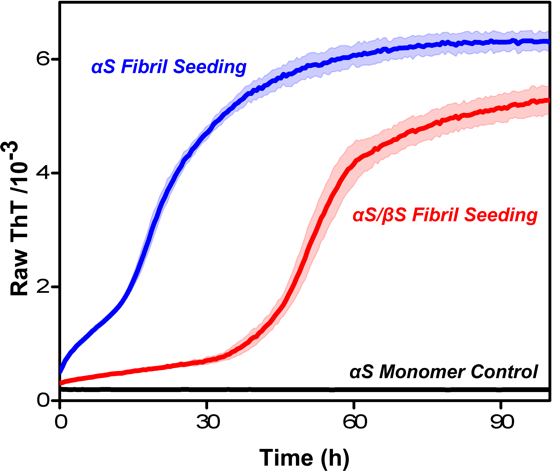


**Figure S3.** ThT fluorescence assay of seeded aggregation. 0.9 μM preformed αS (blue) or αS/βS (red) fibrils were added to a 70 μM αS monomer solution (10 mM PBS pH 7.4) and incubated at 37°C under quiescent conditions (i.e. no shaking). Under the same quiescent conditions, αS monomers alone (black) do not form fibrils.


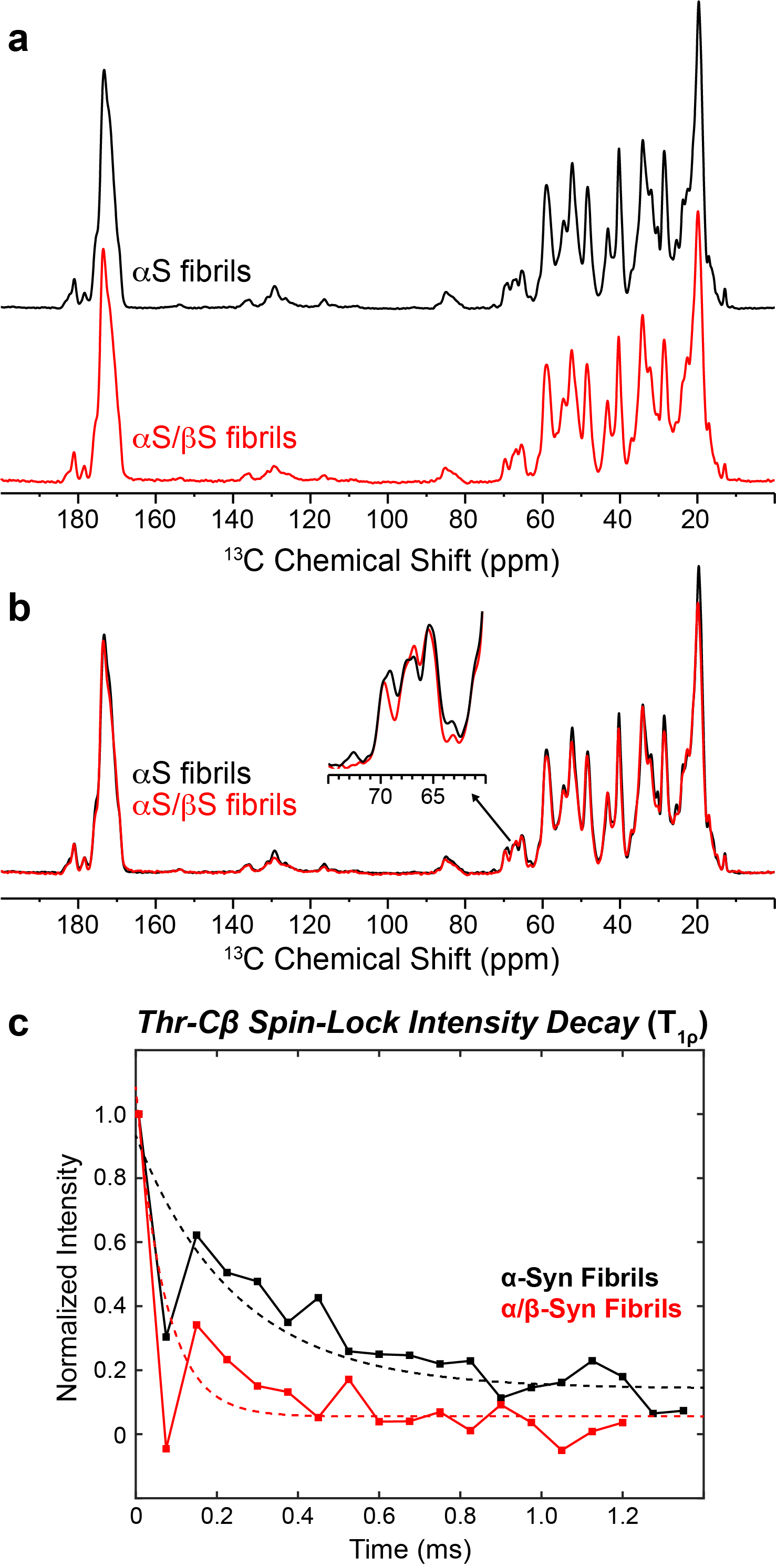


**Figure S4.** (a) One-dimensional (1D) ^13^C cross-polarization (CP) spectra of αS fibrils (black) and co-incubated αS/βS fibrils (red). (b) Overlay of the spectra in (a), with the intensity of the carbonyl region matched. The inset shows the zoomed in region of the Thr Cβ from 60-75 ppm. (c) Intensity decay of the Thr Cβ region under spin-lock, with a ^13^C ω_1_ field of 2.25ω_r_ applied on resonance with the Thr Cβ (70 ppm) during the spin-lock period. Several different ω_1_ rf strengths were tested. The ω_1_ = 2.25ω_r_ condition was found to be optimal, as we observed that it minimized the oscillations of the magnetization due to coherent evolution while providing agreeable RF power levels for our probe. The T_1ρ_ values were observed to maintain their trend for the RF strengths tested. A best-fit simple exponential decay (dotted line) is overlaid to guide the eye. Spectra were recorded at a MAS rate of 13.333 kHz, and temperature was controlled at 25 °C.


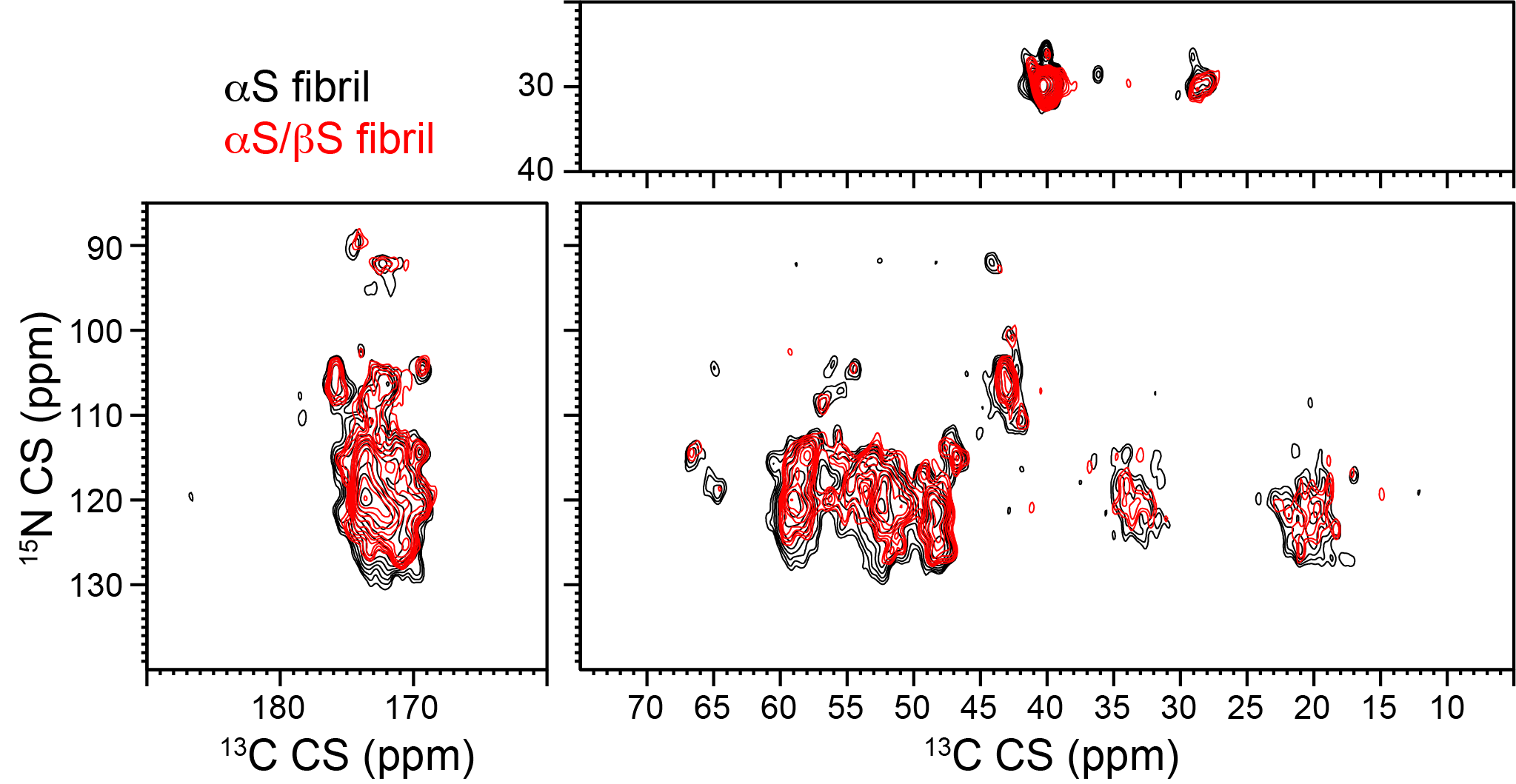


**Figure S5.** Two-dimensional (2D) ^15^N-^13^C heteronuclear correlation spectra of αS fibrils (black) and co-incubated αS/βS fibrils (red). Spectra were recorded using a REDOR based pulse sequence with a REDOR period of 1.35 ms, at a MAS rate of 13.333 kHz, and temperature was controlled at 25 °C.


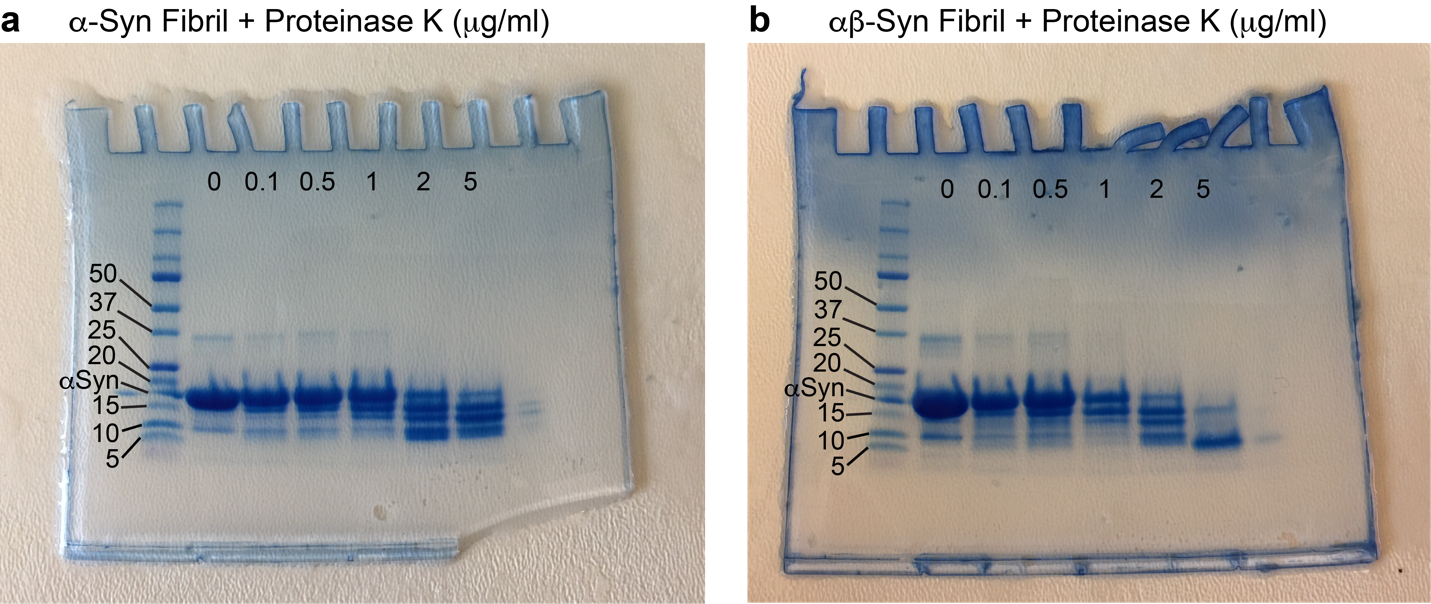


**Figure S6.** Full-length, un-cropped gel images from main text Figure 2c. (a) Digestion of αS fibrils at various concentrations of proteinase K. (b) Digestion of αS/βS fibrils at various concentrations of proteinase K.


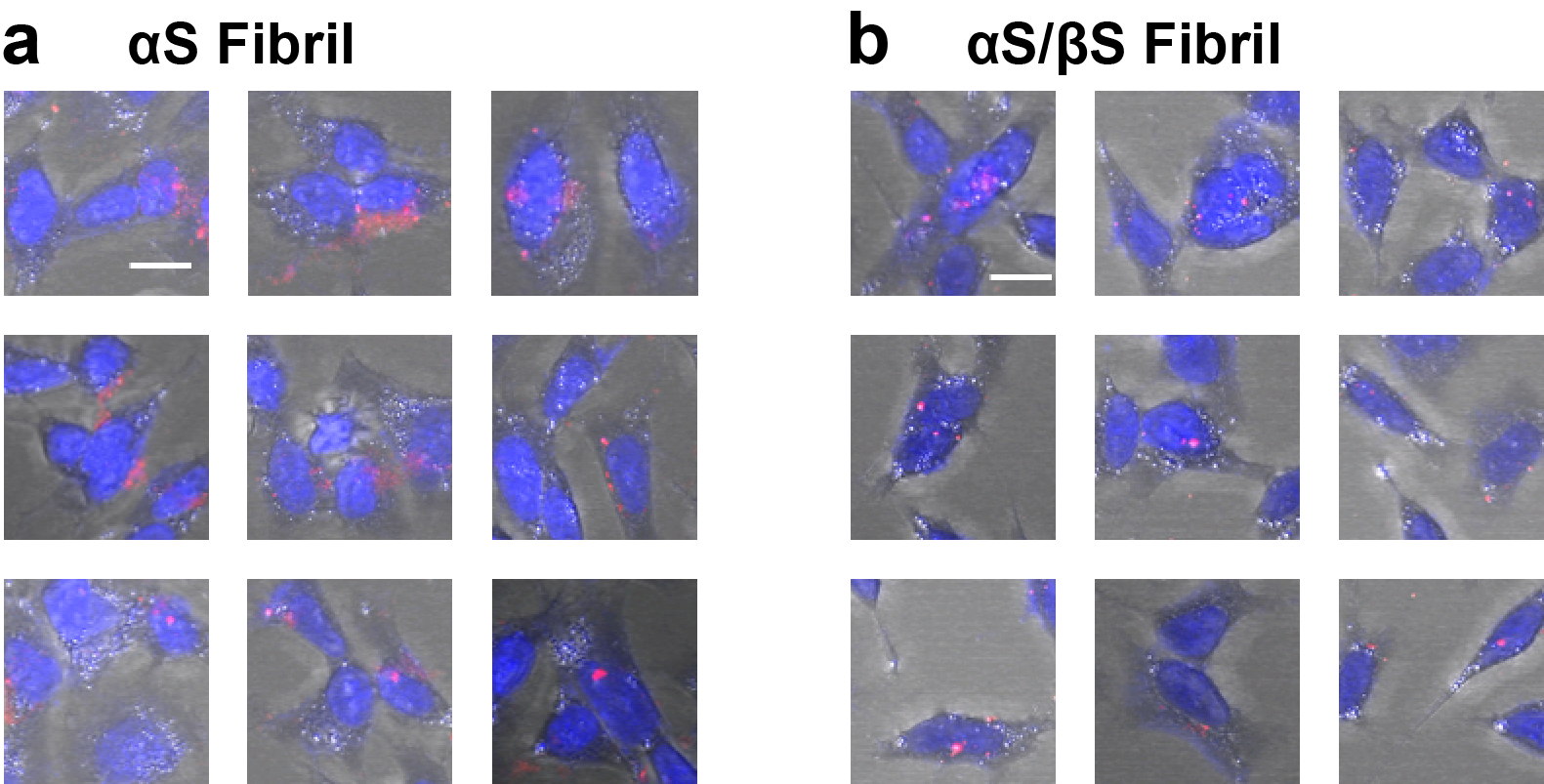


**Figure S7.** Confocal images showing internalized (a) αS fibrils and (b) co-incubated αS/βS fibrils into SH-SY5Y cell. Fibrils were made as described in the main text, labelled with ATTO-550 (red), and incubated with SH-SY5Y cells for 24h followed by fixing and staining with DAPI (blue).


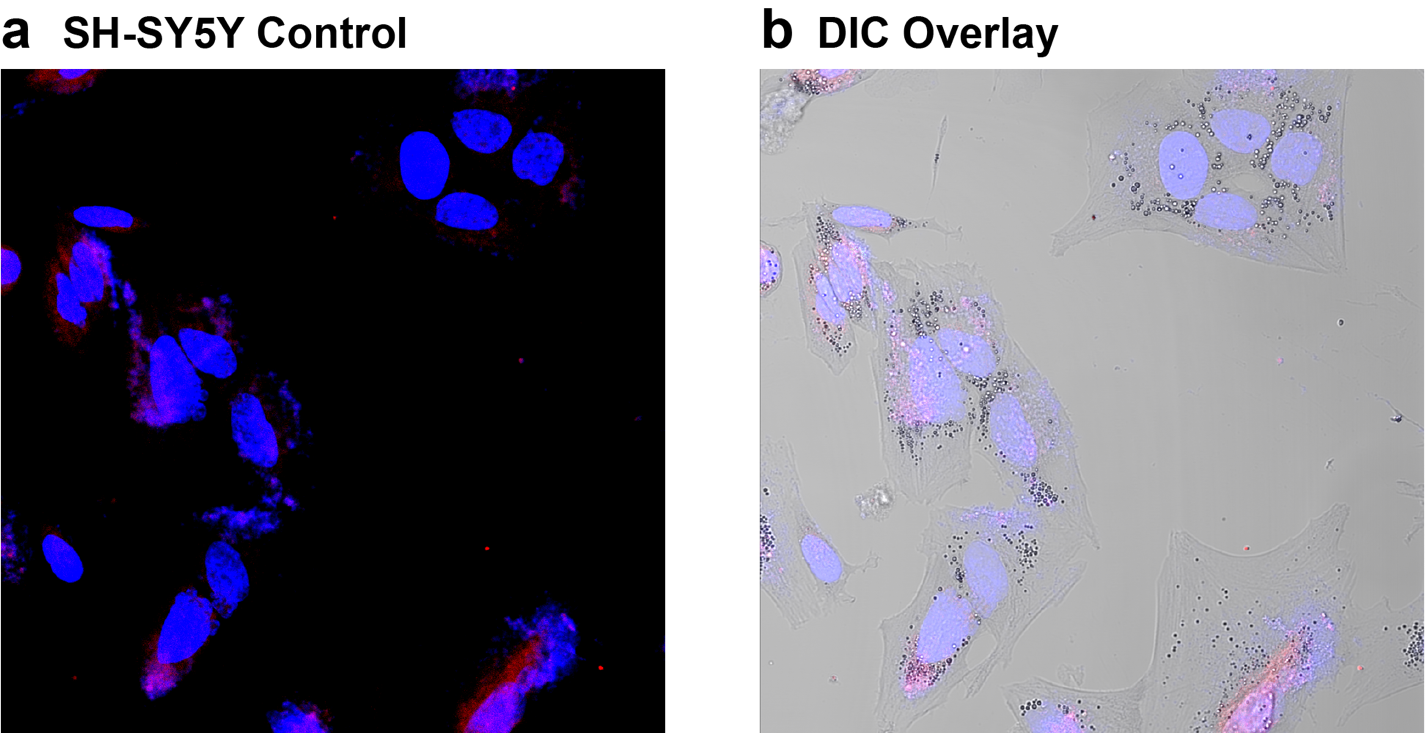


**Figure S8.** (a) Confocal images of untreated SH-SY5Y cells, stained with DAPI (blue) and anti-αS-antibody (red). (b) The same image overlaid with a DIC image of the same field of view.


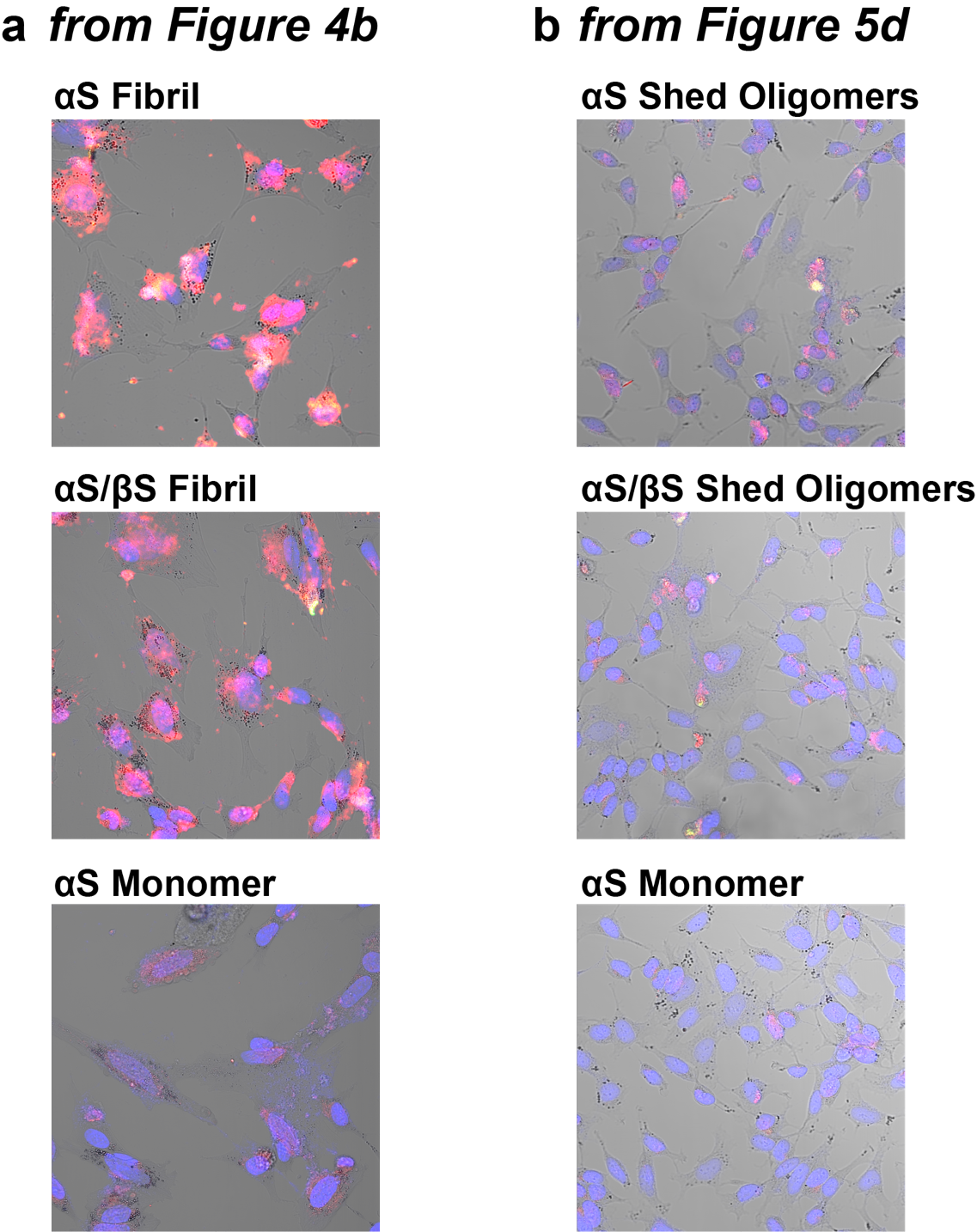


**Figure S9.** DIC images of all cell images presented in the main text of the manuscript. (a) DIC overlays of the merged fluorescence images from Figure 4b. (b) DIC overlays of the merged fluorescence images from Figure 5d.
